# Laugh Away the Fat? Therapeutic Humor in the Control of Stress-induced Emotional Eating

**DOI:** 10.5041/RMMJ.10141

**Published:** 2014-01-21

**Authors:** Elizabeth S. Bast, Elliot M. Berry

**Affiliations:** 1 University of Pennsylvania Perelman School of Medicine, Philadelphia, PA 19104, United States and; 2 Department of Human Nutrition & Metabolism, Braun School of Public Health, Faculty of Medicine, Hebrew University-Hadassah Medical School, Jerusalem, Israel

**Keywords:** Emotional eating, humor, obesity, sociotype, stress

## Abstract

This review explores the potential overlap between the fields of nutrition and therapeutic humor, together with the role of humor as a possible tool for aiding those in whom emotions, particularly negative ones, trigger eating as a means to improve mood. We review emotional eating, obesity, and the hypothesized mechanisms of emotional eating. We then review the field of therapeutic humor and its ability to de-stress individuals, possibly through endorphin and opioid systems, both of which are also involved in eating behavior. Finally, we present a novel hypothesis that people may be trained to use humor as a “food substitute” at best, or to blunt hunger stimuli, to achieve similar advantages, without the side effect of weight gain.

## INTRODUCTION

“You are what you eat” goes the old adage, and the molding influence of nutrition is becoming ever more clear—and with it the concept of the sociotype which strives to understand the importance of an individual’s relationship with his or her social environment and the effect it may have as a determinant of health and body weight.^1^

It is clear that many of us live in an obesogenic environment; consequently average BMIs are trending upward. Obesity is the pandemic of modern civilization and is responsible for the increase in non-communicable diseases worldwide.^2^ Increasing healthy behaviors should be a high priority for health care professionals; however, new tools are needed to combat the ever present allure of the obesogenic lifestyle.

In this exploration of the literature, we propose a new area of investigation between the fields of nutrition and humor, which have not been associated before. In his seminal investigation on humor and laughter, Robert Provine makes no reference to eating, food, or even wine and their effects on mood or humor.^3^ We propose that the phenomenon of emotional eating and the therapeutic potential of humor overlap in the domain of stress activity and management. Furthermore, we hypothesize that new tools and strategies may be created to help those who struggle with emotional eating. We include suggestions for future studies that might be undertaken to investigate this possibility further.

A fruit is a vegetable with looks and money. Plus, if you let fruit rot, it turns to wine, something Brussels sprouts never do.

(P.J. O’Rourke (1947–): *The Bachelor Home Companion*; 1987)

## EMOTIONAL EATING

Despite the plethora of fast food chains, convenience stores, and vending machines providing calorie-dense food in today’s “obesogenic environment,” it is also clear that not everyone is affected by the pandemic.^4^ The question as to why some people remain lean (the so-called “positive deviants”) while others become large is a complicated combination of genetic, environmental, and psychological factors probably best explained by a biopsychosocial and sociotypic model. The concept of “emotional eating” falls within this model.

From an academic perspective, the origin of this concept comes from Kaplan and Kaplan’s psychosomatic theory of obesity which postulated that due to the anxiety-reducing effects of eating, people learned to eat when anxious, resulting in compulsive eating and obesity.^5^ Bruch later theorized that obese people had faulty hunger awareness and had incorrectly learnt the signals for hunger, and that they thus felt the same if they were hungry or uncomfortable emotionally, causing them to eat in both situations.^6^

It has become clear that not all obese or overweight people are emotional eaters, and that not all emotional eaters are overweight; however, the concept remains an important aspect in understanding why some people eat well beyond their caloric needs.^7^ The Dutch Eating Behavior Scale^8^ and the Emotional Eating Scale^9^ have both become useful questionnaires to help tease out “emotional eaters” from “normal” and “restrained” eaters. Those who score as “emotional eaters” consume greater amounts of “palatable” sweet, high-fat foods in response to emotional stress than so-called non-emotional eaters.^10^ Studies have shown that these self-identified emotional eaters may try to regulate the negative emotions caused by everyday life through eating behavior.^11^–^12^ For example, chocolate has been found to lead to an immediate mood increase that is more pronounced among “emotional eaters” than those who score within the normal ranges of these scales.^13^

Most research in the field of emotional eating has focused on negative emotions, especially stress. However, it is of interest that while emotional arousal may increase food intake, with negative emotions more often leading to “comfort foods,” positive emotions may result in a greater tendency to consume healthier foods.^14^–^16^ This area warrants further research. For the purposes of this review we focus on the effects of negative emotions and stress as they relate to obesity.

### Emotional Eating and Obesity

Being overweight is neither necessary nor sufficient for classification as an “emotional eater.” As might be expected, however, rates of emotional eating during negative emotional states are reported to be higher among groups of overweight individuals as compared to healthy-weight individuals.^17^–^20^ For this reason, much of the research on emotional eating has focused on overweight and obese subjects, including bariatric surgery patients. Among this latter group, emotional eating is a common problem and may affect weight loss outcomes.

In a study of 178 pre-surgical bariatric patients, Walfish^21^ reported that 40% of patients subjectively felt that there was an emotional cause involved in their weight gain, while around 40% felt that there was not. Amongst the 40% for whom emotions were causal, stress, boredom, and depression were the emotions most strongly implicated. Given the high rates of emotional eating amongst obese bariatric surgery patients, various studies have begun to investigate differential outcomes based on emotional eating status^22^ as well as pre-surgery coping strategies.^23^ Results have been inconclusive, partly due to the retrospective nature of the studies combined with the relatively short follow-up times given the characteristic extreme fluctuations in weight post-surgery. A shared conclusion of these studies is the importance of pre-emptively identifying those patients for whom emotional eating was a cause of their obesity, and developing programs to foster healthier coping strategies in order to help prevent relapse a year or two down the road.

In a study of Latino adolescents, Nyugen-Rodriquez and colleagues^24^ concluded that it is crucial to help vulnerable adolescents develop adaptive coping skills rather than turning to food when faced with stress, to prevent development of poor lifelong eating habits. There is also an ongoing debate as to whether or not obesity is a form of food addiction.^25^

### Mechanism of Emotional Eating

In order to understand the best coping strategies and behavioral modifications to overcome emotional eating, a better appreciation of the phenomenon itself is warranted. There are two hypotheses, both of which may contribute to the ultimate outcome of mood regulation: nutrient-dependent effects and hedonic effects. In the former theory, mood-modulating effects depend on the specific quality of the food and possible biochemical effects that may occur due to these qualities. In the latter theory, mood is regulated due to the pleasure–reward pathway being activated by the brain, which has become conditioned to enjoy palatable foods, often high in sugar and fat.

#### Nutrient-Dependent Effects

There is much research on the correlation between rates of depression and intake of protein and fatty acid; however, the connection between mood and carbohydrate intake is perhaps most relevant. Experimental diets high in carbohydrates were associated with a better mood than high-protein diets,^26^ and a carbohydrate-rich drink reduced depression in those with premenstrual syndrome.^27^ This fits in well with the theory of increased intake of “palatable” food during emotional eating.^10^

The “Wurtman hypothesis”^28^ postulated improved mood after carbohydrate consumption due to increased tryptophan crossing the blood–brain barrier, resulting in higher serotonin levels. However, this theory has recently been called into question now that it has been shown that <5% of calories in the meal can be from protein in order for tryptophan to increase significantly. This ratio is not common, even amongst such “high-carbohydrate” foods as chocolate and bread—the protein levels are simply too high.^29^ Furthermore, the food must be taken in isolation, after all of the previous meal’s protein has left the gut.^30^ Thus while a soda may indeed affect mood after several hours of fasting via this mechanism, this theory fails to explain emotional eating adequately in general.

Taking an opposite approach, there have been suggestions that carbohydrates may improve mood through reduction of hypoglycemia. Research among starvation victims also showed large increases in irritability, anxiety, and mood swings,^31^ and in the laboratory setting insulin clamp-induced hypoglycemia may result in a tense tiredness state in non-diabetic subjects, perhaps due to hypoglycemic activation of the autonomic nervous system in an attempt to return to euglycemia.^32^ A thorough review of this field of research by Bolton^33^ has shown higher rates of aggressiveness in studies of Quolla Indians, violent offenders, and college students for those individuals who more readily entered a hypoglycemic state during a glucose tolerance test, or who had generally poor glucose control.^33^–^36^ Taken collectively, these studies suggest that hypoglycemia may indeed affect mood, and glucose intake would most quickly return blood glucose to normal, thereby elevating mood. While it has been noted that true reactive hypoglycemia is quite rare as the body controls blood glucose levels very carefully,^37^ Donahoe and Benton have shown that very low blood glucose levels are not necessarily associated with greater aggressiveness.^38^ Perhaps most promising are studies among children^39^ and adolescents,^40^ which have shown decreased irritability and frustration when playing an impossible computer game if given a glucose drink; these changes were observed rapidly. Without more evidence it is difficult to reach any conclusions except that the relationship between insulin release and the propensity for emotional eating should be studied further.

#### Hedonic Effects

Theories of obesity often revolve around the disruption of control of a “set point” which may be located in the hypothalamus,^41^ but may perhaps have evolved only to deal with the more common historic problem of undersupply rather than surplus.^42^–^45^ In recent years several gut hormones have been discovered and shown to control a significant amount of hunger and satiety signaling.^46^ Disruptions in leptin signaling, for example, may lead to obesity, but a genetic defect in this pathway is rare.^47^ Recent studies have combined various study designs with neuroimaging in attempts to elucidate pathways further and understand patterns of eating behavior. More complex systems postulate the regulation to be beyond the hypothalamus, including the pleasure–reward system.^48^ Activation of the mesolimbic dopamine system^49^,^50^ and increases in dopamine in the nucleus acccumbens (the brain’s reward center), upon consumption of palatable food,^51^–^53^ certainly support this theory. Carnell et al.^54^ recently reviewed this literature, including emotional eating. Emotional eating was shown to represent a different neural process than restrained eating and is hypothesized to occur via a dopaminergic response seen on neuroimaging studies to gustatory and olfactory cues.^55^ Additionally, Bohon et al.^56^ used fMRI to examine a group of girls, divided into “emotional eaters” and non-emotional eaters, for responses to the idea of drinking a milkshake while in a negative or neutral mood. The emotional eaters showed greater activation in the parahippocampal and anterior cingulate in anticipation of the milkshake, and greater activation of the left caudate nucleus and left pallidum on actual receipt of it, versus a control tasteless solution. By contrast, non-emotional eaters showed decreased reward region activation during a negative mood. These results indicate a general activation of the reward center, indicating perhaps that emotional eaters have a greater sensitivity in their reward centers during negative emotional states. However, the lack of activation of emotional areas may indicate that while increased reward may promote a tendency to binge, food does not necessarily decrease the negative affect.

In addition, these effects are frequently related to palatability and so-called “comfort foods” which are often high in sugar and fat. Chocolate is well known as a food that people crave. Macht and Mueller showed that there is an immediate response in mood when subjects were given a palatable chocolate (of their choosing). This dependency of the response on palatability and immediacy suggests that the dependency is not due to specific components of the chocolate, but rather a conditioned response. Furthermore, these results were correlated with emotional eating: respondents with higher emotional eating scores showed greater mood change effects.^13^

These changes are hypothesized to occur via endorphin release, since spontaneous eating increases the release of beta-endorphins in rats,^57^ and beta-endorphins are known to inhibit GABA and thus cause an increased release of dopamine. This theory is also supported by the observation that opioid antagonists decrease feeding behavior in rats^57^ as well as thinking about food, feelings of hunger, and preference for sucrose in humans.^58^ Thus overall, while the exact mechanism remains to be elucidated, there is a large body of evidence that supports the theory that eating involves the pleasure–reward system of the brain, and that this may pathologically become dysregulated in “emotional eaters.” The role of the endocannabinoid system is also relevant both in maternal bonding and later food preferences.^59^

### Emotional Eating and Stress

As previously noted, stress has been well documented as a key negative emotion involved in emotional eating.^21^ Oliver et al.^10^ recorded an increase in consumption of high-sweet/fat foods pre-public speaking, widely considered to be a stressful event. Stress caused by an ego-threatening Stroop color-naming task, in which participants determine the color of “ego-threatening” words on a computer screen (e.g. worthless) versus neutral words, has been shown to enhance intake of chocolate among females.^60^ Ego-threatening stressors are also generally associated with the intake of highly palatable, often high-calorie, foods.^61^–^64^

Dallman and colleagues^65^ theorized that comfort food intake may reduce stress by acting on the hypothalamic–pituitary–adrenal (HPA) axis. In rats, higher cortisol levels were found to increase comfort food intake, while chronically high glucocorticoids increased the salience of pleasurable activities. They hypothesized that this mechanism was related to depression in humans: “atypical” depressives gain weight, but maintain normal levels of cerebrospinal fluid (CSF) cortisol, while “melancholic” depressives have increased cortisol. Atypical depressives may experience hyperphagia in order to reduce the activity of their stress network. Thus, the hedonic effects of comfort food may be augmented by subsequent endocrine effects, especially in persons experiencing high levels of stress. On the contrary, Wallis and Hetherington^66^ suggest that stress-related eating is not an effective coping mechanism. Studies have shown that eating does not serve to reduce distress during, or after, eating.^64^,^67^ Furthermore, consumption of “forbidden” highly palatable food may also cause post-consumption guilt resulting in negative affects and undoing any positive changes that may have occurred, especially among women.^68^,^69^

Whether or not eating represents an effective coping mechanism for stress in terms of elevating affect, two facts remain clear: one is that emotional eating is a real phenomenon and is present in a large portion of the overweight population; second, this coping mechanism is not a healthy one for most of those who use it. Emotional eaters who struggle to remain at a healthy weight need help to modify their behavior into healthier patterns.

Large, naked, raw carrots are acceptable as food only to those who live in hutches eagerly awaiting Easter.

(Fran Lebowitz (1946–): *Metropolitan Life*; 1978)

## HUMOR—A TOOL FOR COPING

While the philosophy of humor is ancient, its scientific study is relatively new. The psychology of humor and the beginning of earnest scientific investigation into its therapeutic potential is often attributed to Norman Cousins, author of *Anatomy of an Illness.*^70^ He credited his “miraculous” recovery from ankylosing spondylitis to a self-prescribed treatment of large doses of vitamin C and deep belly laughter; he famously claimed that 10 minutes of belly laughter gave him two hours pain-free sleep. Subsequent research has shown evidence for positive effects of humor and laughter on the cardiovascular system, as an analgesic, and to boost the immune system—in addition to being an effective stress reduction coping mechanism. However, many of these studies have methodological problems, and further research is required in all areas to develop a fuller understanding of the effects of humor on health.^71^ While all these therapeutic effects could also be linked with nutrition, we will focus here on humor and its potential for stress reduction and as a coping mechanism in relation to emotional eating and behavior modification.

In his review of evolution as a theoretical paradigm, Caron notes that humor and laughter are uniquely human, universal traits.^72^ Why humans laugh is a question that has puzzled many. A popular theory for understanding humor includes its evolution as a relief of nervous energy, potentially making it an ideal antidote for stressful situations. This is supported by the empiric observation that mirthful laughter decreases serum levels of cortisol, epinephrine, growth hormone, and 3,4-dihydrophenylacetic acid (a major dopamine catabolite), indicating a reversal of the “stress response.”^73^ “Emotional eaters” who rely on food for mood stabilization develop a maladaptive coping response. We propose that humor may be tried as a new tool in the therapeutic arsenal for those who are dependent on food to manage their moods, or who have pathologic eating habits such as binge eating disorder. Humor has been shown to be a useful coping strategy, and, like hedonic eating, is hypothesized to be associated with the release of endorphins,^74^ although as Martin points out in his review few of the studies comparing pre- and post-comedy exposure have shown significant changes in levels of beta-endorphin. Other benefits of humor may also include a reduction in boredom, which may be another major cause of non-metabolic physiologic eating.^21^

At the outset it should be noted that “humor” is difficult to define and even more difficult to measure. It is beyond the scope of this review to delve fully into the intricacies and caveats of these issues as we are more interested in the general mechanisms, and possible benefits, of humor in respect to eating behavior; however, a brief overview is helpful in understanding the research. Two of the most widely used instruments are Martin and Lefcourt’s Situational Humor Response Questionnaire (SHRQ) and Coping Humor Scale (CHS);^75^,^76^ their usage was reviewed after 10 years by one of the authors, Martin.^77^ The SHRQ was created in 1984 to assess the stress-moderating effects of “sense of humor,” in other words humor as a “trait” in one’s personality. Martin noted that at that time, the only self-report scales in existence were those of humor appreciation. In the process of creating the SHRQ, he stated: “we defined sense of humor as *the frequency with which a person smiles, laughs, and otherwise displays mirth in a wide variety of life situations.*” This definition is somewhat controversial as it may not be necessary to display mirth, per se, in order to have a sense of humor; however, the authors wished to take the most atheoretical and behavioral approach. The SHRQ describes 18 pleasant and unpleasant situations to which respondents are asked about their response (Smile? Laugh?). This scale was validated in several ways, including an unstructured interview, interview with a person who knew the responder, as well as studies of humor generation (in which subjects were instructed to make up a humorous monologue in both stressful and spontaneous/unstructured situations). Martin noted that the scale was originally created for testing in Canadian undergraduates; hence, the situations were tailored towards that population. The subsequent translation and use of this scale in other cultures may therefore present problems, and the scale has also been criticized for conflating laughter generation with a sense of humor.^78^ The CHS was created to investigate how subjects used humor specifically to cope with stressful situations and was validated alongside the SHRQ in many studies.^77^ In response to what they viewed as inadequacy of the available scales to encompass “sense of humor,” Thorson and Powell later developed the Multidimensional Sense of Humor Scale (MSHS).^78^ This humor scale, while probably more accurately encompassing what we might consider a “sense of humor,” may also be too broad when measuring or considering the beneficial aspects of humor (see discussion of the study of Finnish police officers, below). Partially for this reason, Martin and colleagues more recently developed the Humor Styles Questionnaire (HSQ), which breaks humor into four broad categories, two of which are hypothesized to be psychologically beneficial (so-called affiliative and self-enhancing humor) and two detrimental (aggressive and self-defeating humor).^79^

Numerous studies have supported the view that humor and laughter are therapeutic for relieving tension and anxiety,^77^,^80^–^82^ although the results are at times controversial and may show gender-specific differences.^83^–^85^ Nezu et al.^86^ reported that a sense of humor reduced stress associated with depressive symptoms, but did not significantly affect anxiety. Moran and colleagues^85^,^87^ also looked into this question and found that while humorous stimuli caused only modest elevations in mood, an important buffering effect was noted when those who viewed sad stimuli were able to use humor to prevent negative affect. A proposed mechanism for this cognitive effect has been described as a cognitive-affective shift created by humor in a threatening situation to decrease the feeling of intimidation and release emotion.^80^ Abel^88^ explores this shift as a part of the larger model for stress proposed by Lazarus and Folkman^89^ in which stress is dependent on the situation plus a person’s appraisal of the environment and ability to cope, which thus incorporates various personality variables. Kuiper et al.^90^ investigated sense of humor as a personality variable in relation to coping with stressful life events and found that those with a greater sense of humor had more positive perceptions of difficult events and were able to distance themselves emotionally from problems. Additionally, Kuiper et al.^91^ and Lefcourt et al.^84^ found that humor appreciation and the coping technique of “distancing”^92^ were positively correlated. Later work showed evidence for humor- and emotion-focused coping strategies such as “minimization” and “reversal.”^81^ Abel found that there were indeed significant correlations between those with high trait sense of humor (measured with MSHS) and their perceived level of stress, though there were no differences in the number of “everyday problems” between groups. In addition, those students with a greater sense of humor were more likely to use “positive coping strategies” (assessed with the Ways of Coping Scale^92^) such as distancing oneself from the stressor or solving the problems causing the stress.^88^

While trait levels of humor appear to be important, positive coping results are not solely dependent upon having a “good sense of humor.” Yovetich et al.^83^ measured anxiety leading up to a stressful event (an electric shock) with heart rate, electromyography, and self-report while subjects were either listening to a humorous tape, a non-humorous tape, or silence and compared results between groups of “high” and “low” trait humor (as measured by SHRQ). They found that low SHRQ subjects had more anxiety prior to the stress, but also received greater benefit from the humorous audiotape than the high SHRQ subjects.

Both humor appreciation and humor generation are aspects of what we consider to be a “sense of humor,” but the latter has been shown to be more strongly associated with effective coping.^76^ The ability to see humor in a situation and create distance may be key to the coping mechanism, as discussed previously. In an experiment by Newman and Stone,^82^ subjects were split by trait (high or low humor) and instructed to watch a soundless stressful video and generate their own narrative, either humorous or serious (control). Although “high trait” subjects had an easier time in generating their humorous narrative, “low trait” subjects experienced the same physiological benefits from the humorous passage versus the serious. The authors concluded that humor generation may be a highly effective coping strategy and is not limited only to those individuals who seem naturally to be “more humorous,” but may be taught.

Finally, while this evidence points towards humor as an effective coping strategy for some people, it should be noted that the evidence is not unequivocal that humor makes one healthier overall. Preliminary studies have shown that while people with a greater “sense of humor” have a greater subjective satisfaction with their health, they are not healthier per se.^93^ In fact a 3-year follow-up study of the Finnish police officers found that those with a greater sense of humor (measured by MSHS) were more obese and smoked more than those without.^94^ However, it is also possible that many of these early studies did not take into account the subtleties of humor, and different styles of humor may be correlated with different levels of emotional well-being. As mentioned previously, this ambiguity was some of the impetus behind the more recent development of the Humor Styles Questionnaire, in an attempt to overcome these problems. Preliminary results indicate that it may be important to choose “healthy” styles of humor that promote positive affect, and that results should be closely monitored.^79^ It also should be noted that humor is being used as part of psychotherapy, for example in the management of depression.^95^,^96^ However, it is not clear whether the humor used needs to be condition-specific.

Parsley / is gharsley.

(Ogden Nash (1902–1971): *Further Reflections on Parsley*; 1942)

### Hypothesis: The Humor Diet?

**Figure 1.**
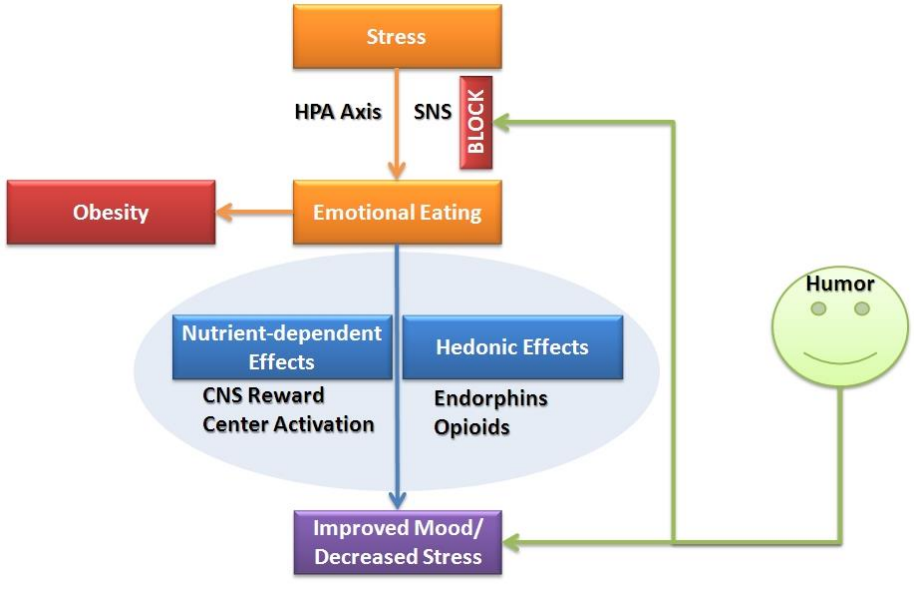
**The Humor Diet Hypothesis.** CNS, central nervous system; HPA, hypothalamic-pituitary-adrenal; SNS, sympathetic nervous system.

Combining these two seemingly disparate fields, we hypothesize that because both emotional eating and humor are intricately related to stress, they may affect each other. Figure 1 provides a diagram demonstrating a simplified mechanism of the hypothesized relationships between these fields, including a model of humor as an alternate pathway to reducing stress. Also included in this figure is the hypothetical relationship between emotional eating and obesity; however, it should be noted that obesity is clearly a multifactorial epidemic, and emotional eating represents only one of a multitude of possible causative pathways.

Future studies to investigate this hypothesis could include designing an active humor intervention, of appropriate “humor style,” and applying it to a group of patients identified as “emotional eaters” who are trying to lose weight, or want to prevent weight gain after bariatric surgery. The intervention could be examined for both humor appreciation and humor generation.

A hypothetical study might be designed as follows: Completion of a self-report questionnaire by a cohort of patients attempting to lose weight to identify emotional eaters; those identified as such would be offered the opportunity of participating in the study. These individuals would then be divided into a control group and an intervention group. In addition to conventional therapy for weight loss used in both groups, those in the intervention group would be trained to identify particular situations in which they find themselves craving comfort food or otherwise “emotionally eating.” They would also be taught specific methods of humor generation. While creating a humorous narrative may not be possible in every situation, there are many ways in which a bird’s eye view and a practiced focus on looking for absurdity might help dissipate stress and calm mood. For example, if stressed about a subject one is studying in school, one could try to come up with silly jokes or puns regarding the subject matter. In addition, although humor appreciation has been shown to be less strongly involved in coping with stress, participants would also be taught to put together a humor “tool-kit,” for example a CD or podcast of a favorite comedian, a book of favorite jokes, or favorite YouTube videos that make them laugh. Participants would use the items in their tool-kit when tempted to snack in a situation recognized as “emotional eating.”

Thus, ultimately, the intervention group would be taught to identify situations causing stress and to use humor instead of food to regulate their dysphoria. Participants would record these situations and uses of humor production and appreciation via journaling. During the study, participants would meet monthly, review their progress, and share any success stories. In addition, a questionnaire developed to investigate the degree to which participants actively used humor as a coping strategy would be given at various intervals throughout the study. A repeat of the original emotional eating questionnaire to assess for changes in ability to control craving would be the primary outcome measurement. Secondary outcomes of interest would include a questionnaire regarding use and success of humor as a coping strategy, data from journal entries, as well as weight loss, and physical activity.

Cauliflower is nothing but cabbage with a college education.

(Mark Twain (1835–1910): *Pudd’nhead Wilson*; 1894)

## CONCLUSION

Given the current understanding of emotional eating and stress, the evidence that humor may be effectively used to reduce stress, even by those for whom laughing off problems does not come easily, and the need for effective coping strategies, we propose humor to be the new *régime du jour*. Despite the complexity of eating behavior, because emotion may play such an important role in people’s eating habits and behaviors—especially if they are “emotional eaters”—we hypothesize that influencing the way emotions and anxiety are managed could have positive effects on eating behavior. Humor has been shown to have numerous positive physiologic effects, one of the strongest of which is in helping people cope with stress. We therefore hypothesize that strategic and purposeful use of humor may provide a useful tool for those individuals in whom stress and anxiety trigger eating of highly palatable foods. This hypothesis is not yet tested; however, we believe it to be one that merits further investigation as it could provide a useful and flexible new tool in the arsenal of those individuals struggling to maintain or return to a healthy weight.

Because this area has not yet been investigated, this hypothesis needs rigorous scientific investigation. Furthermore, it should be noted that there are several caveats inherent in this research. The use of self-report questionnaires in the study of both emotional eating and humor is subject to bias. The cross-cultural effects of humor have not yet been well studied, but must of course be taken into account. In addition, the promotion of other coping strategies, especially those that involve physical exercise, is extremely important to the health and habitus of “emotional eaters,” but perhaps less practical for those times in the middle of the working day when people may feel overcome with stress but unable to leave their desks. In summary, obesity is a multifactorial condition of epidemic proportion across much of the developed world and for which treatment is disappointing. We suggest that humor be investigated as an additional therapy especially among obese people with stress-induced emotional eating problems.

## Abbreviations:

CHS: Coping Humor Scale;
CSF: cerebrospinal fluid;
HPA: hypothalamic–pituitary–adrenal;
HSQ: Humor Styles Questionnaire;
MSHS: Multidimensional Sense of Humor Scale;
SHRQ: Situational Humor Response Questionnaire.
